# Decision-making framework for response and management of environmental disasters (FRaMED)

**DOI:** 10.1007/s00267-026-02502-4

**Published:** 2026-06-19

**Authors:** Wendy K. Bragg, Christy A. Bell, Steve I. Lonhart

**Affiliations:** 1 Department of Ecology and Evolutionary Biology, University of California, Santa Cruz, Santa Cruz, CA USA; 2 Monterey Bay National Marine Sanctuary, National Ocean Service, NOAA, Santa Cruz, CA USA

**Keywords:** Environmental disaster, Emergency response, Decision-making framework, Resource management, Endangered species

## Abstract

Climate change, episodic extreme events, and human-derived non-climatic stressors pose a large threat to natural resources, particularly for niche specialists and species in decline or near range limits. Consequently, natural resource managers must be responsive to environmental disasters, strive to minimize impacts to natural resources, and assess the costs and benefits of management actions. We present two case studies of how managers responded to two different environmental disasters affecting an endangered marine mollusk in California, U.S.A. The first study was a response to a massive landslide, which initially buried some black abalone and their critical habitat, and then slowly expanded up and down the adjacent coastline. The second study was a response to multiple debris flows, generated when a wildfire was quickly followed by an atmospheric river, which also directly buried black abalone populations at watershed outlets and then expanded up- and down-coast. From these two environmental disasters, we developed a suite of materials to facilitate future response operations, including the Framework for Response and Management of Environmental Disasters (FRaMED) presented here. FRaMED was intentionally designed to broadly apply to myriad resources (e.g., species, habitats, ecosystems) and environmental threats (e.g., wildfires, debris flows, oil spills). Moreover, FRaMED is purposefully flexible, enabling managers to adjust actions during and between environmental disasters. Finally, FRaMED can support managers in one-off disasters or in developing detailed response materials for recurring disasters.

## Introduction

Environmental disasters driven by climate change are increasing in frequency and intensity, impacting terrestrial and marine systems alike (e.g., Doney et al., 2012; IPCC 2012, 2013, 2019a, b; Henson et al., 2017; Williams et al., 2015). These long-term, broad-based global trends are punctuated by single or compound extreme climatic events (ECEs) that are increasingly frequent and potentially inflict dramatic ecological impacts (IPCC 2012; Zabin et al., 2022) and drive ecosystem change (Helmuth et al., 2006; Coumou and Rahmstorf 2012; Diffenbaugh et al., 2017; Hallett et al., 2018; Parmesan et al., 2000; Zabin et al., 2022). In addition, human-derived non-climatic stressors (e.g., overharvesting, pollution, invasive species) are also increasing (Geldmann et al., 2014; Schug et al., 2023; Syvitski et al., 2022; Venter et al., 2016). These threats, coupled with background natural disasters (e.g., the 2016 New Zealand earthquake that lifted the coastline 6 m (Gerrity et al., 2020)) increase the potential threat that environmental disasters pose to natural resources. This increased risk is especially elevated for resources that are already in decline or are otherwise rare (Foden et al., 2019).

Calls for adaptive co-management strategies seek to improve preparations and responses to growing environmental challenges for human and natural communities (Månsson et al., 2023; Thorne et al., 2024). It is becoming increasingly important for natural resource managers to address environmental disasters to protect natural resources and mitigate environmental losses. In some cases, injury assessment (assessing impacts to target resources) may be the only option. However, intervention may be warranted for certain target resources and in certain situations, particularly for threatened or endangered species, and where logistically feasible. While distinct specialized response infrastructure exists in limited, well-defined circumstances (e.g., oil spills), the growing frequency and variety of potential environmental disasters present unexplored and expanding challenges, many of which lack historically informed response guidance.

The rocky intertidal habitat along the central coast of California, USA, has highly diverse algal and invertebrate communities (Blanchette et al., 2016) and serves as the primary habitat of black abalone (*Haliotis cracherodii*), a critically endangered marine mollusk (74 FR 1937). Indigenous peoples (e.g., the Amah Mutsun and their Ohlone ancestors, Costanoan, Esselen, Salinan, and Chumash) consumed the large muscular foot of black abalone and traded its shell throughout western North America (Vileisis 2020). Massive declines in the southern portion of the species range due to overharvesting and a fatal wasting disease, “withering syndrome” (WS), led to the closure of the fishery in 1993 (Altstatt et al., 1996). While no catastrophic declines due to WS have occurred on the Big Sur coast, WS continued to decimate populations of black abalone south of San Simeon, and they were listed as critically endangered in 2009 (74 FR 1937).

In 2017 and 2021, on the central coast of California, state and federal natural resource managers from the California Department of Fish & Wildlife (CDFW) and the National Oceanic and Atmospheric Administration (NOAA), respectively, faced two separate but similar environmental disasters that ultimately required active interventions to save black abalone. In the first case, after a particularly wet winter season, a massive landslide occurred in spring 2017 (Handwerger et al., 2019), burying 500 m of coastline and temporarily adding 13 acres of new land (U.S. Geological Survey, Pacific Coastal and Marine Science Center 2017) to the state of California (Warrick et al., 2019). In the second case, an extreme wildfire event in 2020 (Porter et al., 2020) was coupled with an atmospheric river event in 2021, dropping up to 38 cm of rain in 36 hours (NOAA 2021), and generating multiple, simultaneous debris flows within the wildfire scar (Olsen et al., 2024). In both instances, rocky intertidal habitat at the base of steep mountains was abruptly buried by tons of terrigenous unconsolidated material that later expanded the footprint of damage well beyond the initial impact sites. In both cases, teams were deployed to assess the status of endangered black abalone. Real-time examination of the initial and subsequent site-level conditions suggested that intervention to remove affected black abalone could reduce further losses in the face of ongoing and uninhibited habitat destruction.

**Fig. 1:**
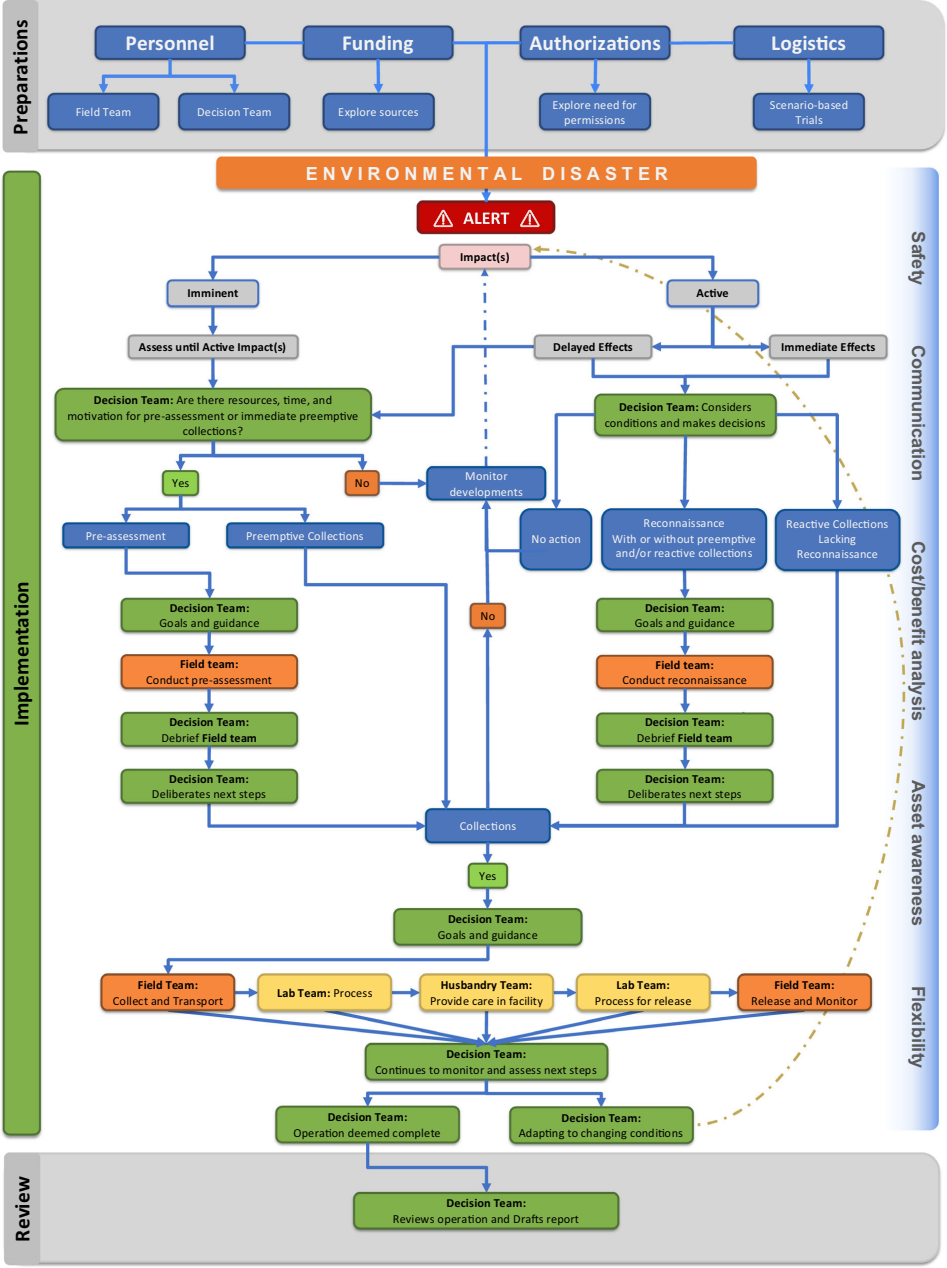
Framework for Response and Management of Environmental Disasters (FRaMED). Note that the Decision Team may decide to pursue one course or a combination of simultaneous or tandem actions, including pre-assessments, reconnaissance, collecting reactively and/or preemptively, leaving the resource in situ, conditionally collecting on a case-by-case basis based on minimization of harm, processing goals and procedures, immediate release vs. holding in captivity, monitoring, and many other options

A post-hoc review of the 2017 and 2021 responses suggested that both natural resource managers and onsite responders would have benefited from a clear and well-developed decision-making framework. Here we introduce the Framework for Response and Management of Environmental Disasters (hereafter FRaMED - Fig. 1) that evolved from direct experiences with these two natural disasters (described in Case Studies 1 and 2, hereafter CS1 and CS2) and was later refined and guided by response materials for oil spills (Oil Spill Response Plans 2024; Responding to spills and pollution 2025) and other emergencies (De Meyer and MacRae 2020; Monterey County Emergency Operations Plan 2020), to produce a generalized tool that is equally applicable to a variety of target resources and environmental disasters. Moreover, the structure of FRaMED encourages adaptability, both during an active response and over repeated application to multiple disasters via iterative adjustments. Thus, FRaMED is designed to meet the changing and diverse needs of natural resource managers within and between environmental disasters.

FRaMED prepares managers to confidently make decisions and implement actions leading up to and during environmental disasters. We present FRaMED in four formats: narrative, flowchart, supporting materials, and case studies. The narrative overview and associated flow chart outline the structure of FRaMED and the activities associated with each phase. More detailed considerations, actions, and responsibilities can be found in Supplementary Materials (A) Decision Guidance, (B) Example Field Reconnaissance Datasheet, and (C) Overarching Considerations. The two case studies serve as real-world examples of the application of FRaMED at a species level, highlighting both its strengths and adaptability. Key terms are defined in the Glossary.

## Material and methods

FRaMED (Fig. 1) is flexible in scale and context, and guides response decisions in preparation for and immediately following an environmental disaster. FRaMED accommodates preemptive and reactive actions and is applicable to multiple levels of ecological hierarchy, from species to ecosystem. FRaMED consists of three integrated phases: Preparation, Implementation, and Review. Additionally, key overarching considerations that underlie all aspects of responses include safety, communications, cost/benefit analysis, asset awareness, and flexibility. See Supplementary (C) Overarching Considerations for a more detailed discussion. Here we present and define the individual components of FRaMED, and then in the Results section, we implement FRaMED in two separate case studies.

### Preparation Phase

Personnel, funding, authorizations, and logistics are most effectively considered prior to an active disaster because they are time-consuming and are likely to determine the feasibility of mounting an emergency response. While attempting to anticipate every circumstance is neither feasible nor efficient, some general preparation tasks apply universally and yield dividends by improving the response actions and anticipated outcomes.

Ideally, these considerations would be addressed before an actual disaster, but generally this is unlikely. Recognizing that environmental disasters are unpredictable, some or all of the following tasks will need to be completed coincident with the Implementation Phase. However, cursory proactive discussions to broadly define goals, obstacles, and opportunities will facilitate efforts during an active emergency response.

#### Personnel

In well-funded and staffed emergency responses, responsibilities may be divided (e.g., operations, planning, logistics, finance, administration, safety, communications) and assigned to specialized leads (U.S. Federal Emergency Management Agency 2017). The typical natural resource response is unlikely to benefit from similar financial and personnel support. Therefore, we have grouped essential responsibilities into two levels: administration and implementation. In practice, the division of responsibilities will scale with the size of the event.

Administration personnel are responsible for decisions, coordination, communication, finances, and project documentation and hereafter called the Decision Team (DT), which has a designated lead and is preferably composed of one representative from each key management or research entity (e.g., natural resource management agency, local authority, academic research group). Having a consistent and formalized DT promotes informed deliberations, timely communication, and shared goals and expectations. Throughout the emergency response, the DT discusses goals, adjusts guidance and strategies as necessary, and maintains records. When environmental disasters are either chronic or well-anticipated, establishing the DT prior to the next emergency bolsters preparedness and a timely response.

Implementation personnel are responsible for carrying out the directives from the DT. While these trained and experienced personnel are charged with field, lab, and husbandry responsibilities, they are collectively termed the Field Team (FT) for simplicity. Depending on conditions, the DT may adjust FT assignments situationally and adaptively. The FT members likely will be drawn from multiple institutions with varying degrees of oversight, limitations, and risk management. The DT must stay aware of this diversity and ensure that all members are covered by medical and liability insurance, as appropriate, by their own institution. See Supplementary (C) Overarching Considerations for more details.

The DT should establish an Emergency Contact Directory that includes all DT and FT personnel with their associated geographic location, experience/skills, costs, responsibilities, and additional key logistical considerations. This directory facilitates the selection and notification of appropriate personnel once the DT is alerted to a disaster. Additionally, if a formal DT does not exist, a resource manager can use the Emergency Contact Directory as a pool of possible personnel to create a DT.

#### Funding

Funding an emergency response entails both obtaining and managing funds. Ideally, funds would be proactively secured and held in reserve until needed (as is the case for oil spills in the U.S., New Zealand, and other countries, where a well-established and formalized process exists). However, the bulk of environmental disasters will lack pre-established funding and typically will not involve a responsible party. Moreover, advocating for funds to support a speculative future disaster can be daunting, time-consuming, and uncertain. Unfortunately, a lack of timely funds may preclude or severely limit the scope and scale of responses, leaving teams scrambling to identify and obtain rapid funding in the hours to days following an environmental disaster. Thus, the DT will often be juggling scientific, oversight, and funding responsibilities during an emergency response. Proactively developing a list of potential funding sources to contact during a disaster can facilitate the funding task.

#### Authorizations

Accessing sites, monitoring or collecting species, and additional activities may require formal or informal authorizations from one or more entities (e.g., government agency, private landowner). Even during an emergency response, obtaining authorizations may proceed slowly, precluding rapid action. To avoid delays, proactive discussions should address the need and procedures to obtain preemptive authorizations or to expedite reactive authorizations. Similarly, remote sensing technologies such as Unmanned Aerial Vehicles (UAVs) and Unmanned Underwater Vehicles (UUVs) can be especially useful in assessing and tracking conditions of impact zones and can enable the extrapolation of recorded impacts to a larger area. However, they often come with location-specific, diurnal, or seasonal regulations and prohibitions that require consideration.

#### Logistics

If intense or repeated environmental disasters are anticipated, it may be advisable to establish a well-developed logistics program. A key feature includes a tailored Emergency Response Plan, which can be generated by adapting FRaMED to the unique characteristics of the target resource and the anticipated threats. This tailored FRaMED can prepare personnel a priori through scenario-based training to anticipate developing issues and to increase efficiency, effectiveness, and safety during an active emergency response.

### Implementation Phase

The Implementation Phase comprises the bulk of the actions undertaken during a response. This phase includes the initial alert and exploration of risk, decision-making for and execution of action options, which may include collections, and adaptation to changing conditions. Throughout the Implementation Phase, there are several opportunities to refine or modify directives as circumstances change.

#### Initial Alert and Exploration of Risk

The Implementation Phase begins when natural resource managers are alerted to an active or imminent environmental disaster, which has or is likely to affect one or more target resources. While some environmental disasters use warning systems to generate an alert (e.g., tsunami or tornado sirens), in most cases the initial alert will rely on reports from the public, information outlets (e.g., weather forecasts), or observations by field researchers involved with long-term monitoring (LTM) programs, further emphasizing the importance of LTM programs not only as environmental monitors but also as potential warning systems. When available, LTM data can inform injury assessments, urgency of response, and collections (either preemptive or reactive).

Once alerted, the DT assesses whether the environmental disaster poses active impacts or imminent impacts to the target resource(s). Whether active or imminent, the DT explores the associated risks by considering the nature of the disaster, threats posed, and knowledge gaps. Historical knowledge of site characteristics and target resources will facilitate both decision-making and readiness to respond. In conjunction with this early risk exploration, the DT may begin weighing decisions by gauging safety, logistics, resource assessment, predictions, photos and imagery, communications, and sensitive information. The factors influencing decisions and their relative importance vary by the type of target resource and the nature of the environmental disaster. A brief overview of these factors is incorporated into the following narrative, but a more extensive exploration can be found in Supplementary (A) Decision Guidance.

Regardless of the impact type or preferred response action(s), monitoring is an important complementary activity. Monitoring may be conducted at one or more active or imminent sites, assuming it is safe to do so, and may continue for an extended period, even after other response actions have ceased. Monitoring can influence decision-making, increase understanding of disaster processes, and facilitate the analysis of success. The monitoring scope will also be determined by assessment goals. For instance, if injury assessment, with or without collections, is an identified goal, monitoring is likely to include impact site(s) as well as reference sites. However, delays in information acquisition due to limitations in mobilization, data gathering, and analysis can limit the real-time utility of monitoring data in the decision-making process.

#### Impacts

Environmental disasters, which vary in spatial and temporal extent, magnitude, frequency, and intensity, are expected to dynamically impact the target resource. The impact zone encompasses the broadest extent of current and potential future environmental injury. If the impact zone is a mosaic of both undamaged and damaged areas, the damaged areas are designated as impact sites within the wider impact zone. Some impacts may be preceded or followed by an alert. Imminent impacts are preceded by an environmental alert, such as when a volcano emits gasses prior to an eruption. Active impacts present suddenly and unexpectedly, such as a lightning fire or a remote pipeline rupture. Active impacts can have both immediate effects and delayed effects. FRaMED accounts for all these impact scenarios.

##### Imminent Impacts

Depending on current conditions (safety) and the availability of personnel, funds, and authorizations, imminent impacts may afford a grace period during which the FT can access identified at-risk sites to conduct pre-assessments and/or preemptive collections. When pre-assessment is feasible, the pre-impact data can be used to gauge potential risks to the target resource and/or for later comparison with possible post-disturbance data to quantify the extent of injuries incurred. In some cases, preemptive collection of unaffected target resources may be conducted to minimize anticipated losses. Alternatively, the DT may decide to, or be forced to, forgo preemptive collections, which may be the most appropriate (e.g., limited effectiveness) or the only option under certain circumstances (e.g., lacking authorization, site inaccessibility, hazardous conditions, insufficient personnel, lack of funding).

##### Active Impacts with Immediate Effects

Sudden and unexpected environmental disasters preclude both pre-assessment and preemptive collections in active impact sites. Here, the DT considers three general response options: no action; reconnaissance with or without collections; or reactive collections without simultaneous reconnaissance.

No action may be the best option in some circumstances, especially if risks to personnel and equipment are at an unacceptable level, if adequate funds/personnel/equipment/permits are unavailable, if effective intervention is deemed unlikely, or if the DT determines that delaying action may improve the overall outcome.

Ideally, reconnaissance of active impact site(s) is possible to assess current conditions, document current impacts to target resources, and evaluate future risks and the potential for delayed effects. See Supplementary (B) Example Field Reconnaissance Datasheet. Reconnaissance goals and actions are determined by the DT, and reconnaissance data can inform DT decision-making by informing cost/benefit analyses. In some cases, reconnaissance and target resource collections may be combined and completed simultaneously. These opportunistic collections may be reactive, preemptive, or a combination of both (see *Collections* below). Initiation of collections may be prescribed by the DT or may result from an abrupt shift once the FT arrives on-site, with or without consulting the DT. See Flexibility in Supplementary (C) Overarching Considerations.

In extreme situations when site conditions and a sense of urgency dictate (e.g., limited safe operating window), reactive collections may be conducted in the absence of reconnaissance. Here, the sole goal is to collect as much of the target resource as possible as quickly as possible to minimize losses. Reactive collections generally prioritize removing individuals who are experiencing active impacts. However, if conditions suggest expanding or intensifying impacts, reactive collections may be expanded to incorporate simultaneous preemptive collections, particularly in the face of rapidly deteriorating conditions and logistical constraints.

##### Active Impacts with Delayed Effects

Delayed effects from active impacts come in at least two forms: (1) expansion or intensification of known impact effects (similar to existing effects already observed), and (2) secondary unanticipated effects (those of a different character from observed effects). An expansion of immediate effects generates a scenario analogous to the imminent impacts: as the impact spreads beyond the initial footprint, there is a lag before new sites are impacted. These phased impacts may require repeated or extended actions similar to the initial response actions, applied to locations further and further from the initial footprint. Such expansion may enable preemptive collections in areas where impacts are anticipated (see *Imminent Impacts*). In contrast, secondary unanticipated effects are truly novel and may require an entirely different type or degree of response actions. Unanticipated effects essentially present as unique immediate effects (see *Active Impacts with Immediate* *Effects* below), requiring a fresh exploration of risk and consideration of emergency response actions, and may require additional personnel with unique areas of expertise.

#### Decision Team: Goals and Guidance

The DT uses FRaMED to guide the emergency response, selects personnel for one or more FTs, and provides overall guidance. There are multiple considerations when deploying an FT (see Supplementary (A) Decision Guidance), and priorities may change over time as the situation evolves. Certain disasters may warrant such a rapid response that DT deliberations are impracticable. These conditions highlight the importance of having a single designated lead within the DT to initiate rapid action, which may or may not be subsequently followed by more extensive DT discussions as events develop. In such cases, the DT lead’s familiarity with FRaMED may facilitate safe and meaningful operations.

#### Collections

Collections may be reactive, preemptive, or both. Collections may be done alone or in combination with pre-assessment or reconnaissance. Removing individuals from harm and tending to injuries at an off-site facility may prevent or mitigate losses. After the environmental disaster no longer poses a threat, recovered and healthy individuals can be returned to their original locations or translocated to nearby unaffected sites within the impact zone or to suitable sites beyond the impact zone. Alternatively, collected individuals may be slated for retention in a facility (e.g., if not fully recovered or unlikely to survive or if no viable option exists in the wild).

The decision to collect target resources initiates a series of subsequent complicated actions that require special considerations. Important components include: transportation from the field, husbandry in a temporary or permanent facility, possible tagging, and returning or translocating back to the wild. Monitoring individuals is important throughout to assess health during transport and holding, and to track survivorship after return or translocation. These post-collection and post-release monitoring data are crucial to informing future decision-making. If collections of any kind are warranted and have been authorized, the DT should provide additional guidance to the FT outlining procedures for selecting individuals based on condition, relative proportion of the population, and other factors.

#### Adapting to Changing Conditions

FRaMED allows for responsive decision-making and the need to reassess conditions frequently and repeatedly. As conditions change, the initial guidance, goals, and actions may shift in return. Response actions may be repeated, paused, or changed multiple times before the overall response ends. Importantly, per DT guidance, FTs may be simultaneously conducting multiple different actions at one (as in CS1) or multiple impact sites (as in CS2).

### Review Phase

Once an emergency response ends, the DT should review and formally document all stages of the response, describing: the onset and evolution of the environmental disaster; a summary of the preparation and implementation phases; outcomes; lessons learned; and recommendations for future responses, including suggestions to modify FRaMED. The review phase is facilitated by accurate record-keeping during the response by both the DT and FT.

## Results: Case Studies

The following case studies (CS1 and CS2) focus on two sediment-based environmental disasters and their impacts on the endangered black abalone. CS1 occurred prior to any decision-making development, lacked an alert, and included both immediate and delayed effects of a landslide. CS2 included an alert followed by multiple active impact sites with immediate and delayed effects from debris flows. Both case studies also incorporated monitoring of conditions at impacted sites and adjacent areas. These two case studies highlight the importance of pre-planning, adapting FRaMED to specific conditions (even under seemingly similar situations), and maintaining flexibility to ensure optimal outcomes.

### Case Study 1: Impact - Active with both Immediate and Delayed Effects

Overview: In 2017, a sudden massive landslide in Big Sur, California--a rugged stretch of scenic coastline between Carmel in the north and San Simeon in the south--buried approximately 500 meters of intertidal coastline (Warrick et al., 2019) that was designated as critical habitat for black abalone (76 FR 66806). The massive landslide destroyed the single road in the area and triggered action by multiple state and federal agencies to stabilize the slide and repair the highway (Handwerger et al., 2019). Historical data on the abundance and distribution of black abalone were lacking at the impact zone due to its remoteness and inaccessibility, but nearby LTM data suggested black abalone presence. Here, the impact zone and the impact site were one and the same.

#### Preparation Phase

##### Personnel

A pre-existing FT composed of black abalone experts with longstanding monitoring permits and with experience responding to oil spills regularly conducted LTM surveys at sites both north and south of the impact zone prior to the slide. An informal DT consisted of a Principal Investigator (PI) and regulatory agency staff with well-established relationships with the PI.

##### Funding

As a natural disaster, there was no responsible party to contribute to mitigation and recovery funding. The resource trust management agencies (i.e., California Department of Fish & Wildlife, NOAA Fisheries, and Monterey Bay National Marine Sanctuary) and other key organizations, which lacked funds to directly pay for response expenses, instead provided in-kind support by re-assigning staff members to assist with the FT (196 people hours were donated in total by five organizations). Some funds (~$30,000 U.S. Dollars) from a non-resource trust agency (California Department of Transportation) were obtained on an emergency basis, circumventing the normal grant process.

##### Authorizations

As the California Department of Transportation (Caltrans) worked to stabilize the landslide above and below the highway, the FT acquired additional authorizations to use a UAV to monitor site conditions. Existing endangered species permits were in place to handle black abalone, while additional permits were rapidly obtained to collect, transport, and hold black abalone in captivity.

##### Logistics

No formal emergency response plans existed for black abalone when the 2017 landslide occurred. FTs relied on extensive experience with post-oil spill injury assessments, vessel groundings, and deep knowledge of black abalone gained through LTM in the region to guide response actions.

#### Implementation Phase

##### Initial Alert and Exploration of Risk

The landslide was unexpected and occurred months after the end of the region’s particularly wet winter season. Although the region experiences small-scale landslides somewhat regularly in the winter, massive landslides such as this were infrequent. Members of the DT learned of the landslide (alert) almost immediately due to media coverage and emergency notifications from Caltrans. However, it took two months before Caltrans reestablished roads and deemed the impact zone safe enough for access by the FT.

##### Active Impacts with Immediate Effects

Initial on-site response to immediate effects was limited to aerial monitoring by a UAV. Aerial imagery was combined with modeled LTM data to estimate population densities at the site. No further action was possible until the site was safe to access by the FT.

##### Active Impacts with Delayed Effects

Once accessible, the DT directed the FT to conduct reconnaissance at the margins of the landslide to locate black abalone (if present) and document possible impacts to black abalone critical habitat. The FT found hundreds of black abalone in good-quality critical habitat adjacent to the impact zone, ranging in size from 20 to 170 mm (greatest dimension), and most in aggregations >1 abalone/m^2^, which is believed to be the threshold for reproductive success (Miner et al., 2006). The feasibility of removing the black abalone without further damage was assessed in situ and relayed to the DT. These black abalone data were the first ever recorded for the impact site and were essential to inform deliberations on potential collections and translocations.

##### Decision Team: Goals and Guidance

UAV images and reports from Caltrans indicated that wave action was moving the deposited unconsolidated material both up- and down-coast, increasing the impact zone beyond the initial footprint of the landslide. These delayed effects threatened to expand losses beyond those from the immediate effects. Additionally, planned road repairs by Caltrans were expected to further jeopardize black abalone and their critical habitat. Armed with reconnaissance data and projections for expanding impacts, the DT deliberated and ultimately determined that the benefit of collecting and translocating some black abalone outweighed both the certain risk of injury or mortality during collection and the potential imminent risk of burial and death.

##### Collections

The DT considered several preemptive collection options for the at-risk black abalone next to the impact zone, including: (1) collect and immediately translocate abalone far from the impact zone; (2) collect and bring abalone into captivity ( ≤ 24 hours) until tides allowed translocation to a location far from the impact zone; (3) collect, bring into captivity, monitor health, and conduct phased translocations far from the impact zone; and (4) collect and immediately translocate some abalone, and transport the remaining individuals to a captive facility. The DT and FT discussed goals and collection logistics and settled on option #2. The recipient translocation site and temporary captive facility were selected, and an emergency authorization was obtained to collect, transport, hold, and translocate black abalone.

##### Adapting to changing conditions

Collecting black abalone posed several challenges. Typically found clinging tightly to coarse rock surfaces, often deep within narrow crevices and cracks, black abalone are removed by using a specialized metal pry bar to forcibly detach individuals from the rock. Due to the force needed to dislodge abalone, it is common to injure the muscular foot with cuts and pressure or to crack the brittle shell. Because abalone lack a clotting factor, these injuries are often lethal. Despite the skills of expert abalone collectors, a small portion of those collected sustained injuries that were deemed severe and were therefore transported to a laboratory facility in hopes of saving some with treatment. Proactive communication and longstanding relationships with experts enabled a quick shift of disposition for these animals to an appropriate facility where care was provided while they recovered. Over half of the injured individuals ultimately survived with veterinary care, and the knowledge gained improved outcomes in CS2 and subsequent management strategies. Uninjured black abalone were relocated to nearby unaffected habitat.

#### Review Phase

A summary report was drafted that highlighted key developments and pivotal points in the response and served as the first iteration of FRaMED that was used and further modified during CS2 (Bell and Raimondi 2020).

### Case Study 2: Impact—Imminent, Assess Until Active Impacts

Overview: In 2020, California experienced a record-breaking fire year (Porter et al., 2020; Safford et al., 2022). The Dolan Fire burned along 40 km of the central coast for 4.5 months and was extinguished on 31 December 2020 (Porter et al., 2020). The coastline adjacent to this fire scar served as the impact zone. The alert warned of imminent impacts, which only resulted after a January 2021 atmospheric river dropped ~38 cm of rain in two days on the burn scar (NOAA 2021), triggering multiple debris flows (Olsen et al., 2024) that buried several populations of black abalone and their critical habitat, thereby creating multiple impact sites within the impact zone. For CS2, a combination of events triggered the effects, which allowed a grace period for planning: the first event (fire) required a second event (atmospheric river) to generate impacts (debris flows).

#### Preparation Phase

This response benefited from the CS1 Review Phase, which identified several lessons learned and generated some recommendations and planning materials, including key components of FRaMED (Bell and Raimondi 2020).

##### Personnel

The continuity of personnel between CS1 and CS2 provided a foundation that boosted the skillset, knowledge, and speed of response in CS2. Again, no formal DT existed, but an informal decision structure composed of the same PI, FT members, and other key players from CS1 facilitated a rapid emergency response. Experiences with CS1 generated a list of appropriate additional personnel to involve in CS2.

##### Funding

As in CS1, temporary funds supported early response efforts while rapid funding was obtained from non-governmental organizations (National Marine Sanctuary Foundation, The Nature Conservancy) to support the rescue/relocation and post-release aspects of the response. During the 3-year operation, from pre-impact baseline data collection (adjacent to four fires) through post-release monitoring, including cost-sharing expenses, ~$250,000 USD were spent.

##### Authorizations

Overarching emergency response authorizations for black abalone collections were not in place, however, the CS1 experience provided a model for how to obtain specific authorizations quickly during an emergency response.

##### Logistics

The first iteration of FRaMED was developed as an outcome of the CS1 Review Phase. Additionally, CS1 served as a real life “trial run”, providing most personnel in CS2 with hands-on experience.

#### Implementation Phase

##### Initial Alert and Exploration of Risk

While no reports of fire-related effects on black abalone were known prior to CS2, there was a clear record of the propensity of fires to leave landscapes prone to accelerated erosion (DeBano 1981, 2000; Meyer 2002; Cannon and Gartner 2005; Neary et al., 2005; Warrick et al., 2022; NOAA National Weather Service n.d.). Post-fire rain, especially if heavy or prolonged, can generate debris flows (Wieczorek 1987; Cannon et al. 2010; Warrick et al. 2012, 2022; Staley et al. 2017; Alessio et al. 2021), which can mobilize up to 35 times the average downslope sediment (Warrick et al., 2012). The awareness (alert) of possible outcomes from this combination of forces (fire + flood = debris flows) offered a grace period during which to prepare for imminent impacts and to conduct reconnaissance. Such a grace period may or may not occur in other emergencies.

Having identified a possible source of impacts (debris flows) it was unclear which particular attributes might impact the species (e.g., burial, scour, turbidity, soil-borne pollutants, and fire-derived compounds). However, the threat was expected to generate impacts, raising an alert. As winter weather approached, the DT used LTM data to calculate possible exposure at 70-90% of the known U.S. black abalone mainland population (MARINe 2020). FT members combined maps of waterways (ESRI 2010), the Dolan Fire scar (PWFDF Dashboard n.d.), and aerial imagery to identify and rank the most at-risk coastal areas. The DT used this information to explore risks and plan initial actions.

##### Imminent Impacts

Having assessed several potential risks associated with debris flows but still uncertain about future impacts (if any), the DT then focused on tracking imminent storm events and discussing options for emergency collection authorizations if needed. Meanwhile, the DT directed the FT to conduct pre-assessments at as many at-risk sites as possible, recording numerous metrics designed to capture a range of potential impact types.

##### Active Impacts with Immediate Effects

While pre-assessments were still underway, an atmospheric river was forecast to move over the Dolan Fire burn scar. The team braced for impacts and monitored information sources for results. Once active, the volume of material delivered by multiple debris flows exceeded expectations, the immediate effects (known from aerial imagery) were deemed to be devastating, and initially precluded accessing multiple impact sites due to unsafe conditions.

##### Active Impacts with Delayed Effects

Delayed effects were not originally anticipated but were identified over subsequent months and years. See *Adapting to Changing Conditions*.

##### Decision Team: Goals and Guidance

While the teams waited for conditions to become safe and sites to become accessible, preparations for various scenarios were completed. The DT requested provisional emergency collection authorizations that could be rapidly activated if needed, a facility was identified and prepared for holding abalone, and the FT prepared simultaneously for reconnaissance and possible collections.

Once safe to do so, the FT initiated reconnaissance, triaging site visits based on historical data, the risk-level map, safe access, and local reports of impacts. On-site observations immediately revealed impacts to both critical habitat and black abalone that exceeded expectations. Impacts from unconsolidated material were so immediately massive and widespread that concerns of longer-term impacts from toxins, nutrient pulses, and other stressors were deprioritized. The FT documented site conditions and returned to brief the DT, which further modified directives to shift to immediate reactive collections.

##### Collections

Multiple simultaneous impact sites within the impact zone overwhelmed FT staffing and capacity, but pre-disturbance data enabled informed site prioritization. The FT collected displaced and injured black abalone while documenting unusual behaviors, locations, and predation events. Collected abalone were processed and received into the pre-approved facility. Rapid funding from an NGO (National Marine Sanctuary Foundation) was provided to support the more extensive, time-consuming, and personnel-intensive emergency response.

After collecting black abalone (*N* = 213 individuals), the FT focused on husbandry while the DT addressed final disposition considerations. The severity and persistence of impacts precluded returning animals within or adjacent to the impact sites. Additionally, the DT sought multiple recipient sites, both to minimize any site-specific hazards and to accommodate the large numbers of animals that had been collected. With the mosaic of impacted and unimpacted sites nested within the larger impact zone, the FT was able to translocate within the impact zone to unaffected areas and to sites beyond the impact zone. Individuals were translocated to these new sites 5 months after collection.

##### Adapting to Changing Conditions

Throughout CS2, goals and directives evolved in parallel with new information, and the team adapted quickly. Delayed effects were not initially anticipated by the DT, but one year after the atmospheric river event, ongoing monitoring of impact sites revealed that adjacent, unaffected sites were still rapidly deteriorating as sediment transport from the original debris flow footprint moved downcoast. As these delayed effects were identified, the FT conducted an additional, relatively small collection and translocation of black abalone (*N* = 10 individuals).

#### Review Phase

Review of CS2 highlighted the unique features of this event including: a grace period between the fire and the atmospheric river that enabled pre-assessments and logistics discussions to facilitate rapid authorizations and pre-approval of a husbandry facility; a broad impact zone with multiple, nested impact sites; near universal a priori support from scientists, many of whom had gained first-hand experience in CS1; rapid acquisition of funding; and a dedicated full-time coordinator for the emergency response. While each situation will have its own unique nuances, advantages, and challenges, the CS2 review underscores the built-in adaptive capacity of FRaMED to accommodate unanticipated challenges as well as the iterative improvements that can be made with repeated application.

Subsequent discussions acknowledged the likelihood of future environmental disasters, both similar to CS1 and CS2 and of unknown origins, and identified additional areas for improvement. To address these, the DT has broadened preparations to include a 10-year standing federal Emergency Response Permit (ESA Permit #26342); statewide species-specific Emergency Response Plans for non-oil spills (Black Abalone Recovery Team Monitoring and Emergency Response Sub Team (BART-MER) 2025) and oil spills (in preparation); an Emergency Contact Directory; new partnerships with husbandry facilities; expanded database of baseline drone imagery of coastal habitat; Go-Kits for collection, processing, and husbandry strategically stationed across the species’ range; and scheduled scenario-based emergency training. While developed after sediment-based emergencies, two complementary versions of these materials were created: one designed to be widely applicable to known and as-yet-unknown threats and a second tailored to oil spills.

## Discussion

Natural resource managers must diversify their risk assessment and emergency response tools to better protect natural resources from environmental disasters. FRaMED is designed to guide preparation, decision-making, and response activities before, during, and after such environmental disasters. The case studies presented here demonstrate the actual implementation of FRaMED, and we indicate some limitations and the capacity to adapt within the system. Details related to long-term monitoring of impacted sites and of translocated species are beyond the scope of this paper; we recognize that tracking the survival of rescued and translocated individuals is key to assessing the success of the effort and to inform future responses. In addition, our case studies included environmental disasters that had both an abrupt phase, where an immediate impact occurred without anticipation and no time to respond quickly, and a slow phase, where the spread of unconsolidated material progressed over weeks and months, allowing a planned and logistically complicated response, including rescuing black abalone prior to burial, and translocating them beyond the influence of the environmental disaster.

FRaMED is widely applicable to the unique needs of natural resource managers. While some level of proactive preparation or contemplation is ideal, FRaMED can be employed in an impromptu manner if needed. Alternatively, more extensive preparations should be considered for resources that are particularly rare, vulnerable, or subject to frequent or intense environmental disasters. Other circumstances may warrant multiple tailored frameworks for the same region. For example, if models predict that fires and hurricanes are both likely in a region, different response options that uniquely fit each environmental disaster should be created. Similarly, each species of concern may require unique frameworks and emergency responses. Even in systems where some planning and protocols already exist, exploring FRaMED can clarify responsibilities, timelines, important considerations, and identify gaps to address.

FRaMED is designed as a generalized, adaptive tool that can be further refined through an iterative process within and between emergency responses to match the unique needs of natural resource managers. Within an active response, FRaMED encourages repeated reexamination of conditions and shifting of actions as many times as conditions require. Managers can follow FRaMED while incorporating new information, expanding, shrinking, or diversifying actions. This within-response dynamic of the framework is exemplified by CS2: from an alert to pre-assessment, back to monitoring, then to active impacts, reconnaissance, and collections, back up to delayed effects, which prompted a second collection, before finally ending, nearly two years after the initial debris flow impacts occurred. FRaMED is also designed to guide personnel through a post-response review phase (as occurred after CS1 and CS2) that encourages refinement, improvement, and additional preparations for future impacts or new responses. While beyond the scope of FRaMED, post-response monitoring is also necessary to assess the effectiveness of implemented actions, particularly when compared with areas lacking interventions, to better understand natural recovery and whether no action is a viable option for resource managers. For instance, in CS2, resampling of impacted sites revealed 94–100% mortality risk in habitat that was classified as completely buried in sediment and 30–32% mortality in areas within the impact zone that would have been judged “unaffected” based on visual examination of aerial imagery (Bragg et al., 2026). Median losses were estimated at 59.6% of the population within the 40 km impact zone (13,598 individuals). While 213 individuals represent a small proportion (1.6%) of overall estimated losses, the near complete decimation of populations within this important population stronghold for the species provides additional critical information to support FRaMED modifications as managers plan for future environmental disasters.

When faced with environmental disasters, natural resource managers have myriad options, ranging from mounting an immediate, intensive response to taking no action. FRaMED is designed to structure and guide these comprehensive discussions, deliberations, and, if warranted and feasible, emergency response actions. Anticipating an increase in environmental disasters globally, FRaMED was intentionally generalized to be broadly applicable, providing an adaptive and flexible framework around which customized emergency response plans can be designed for application to a variety of target resources, environments, and environmental disasters.

## Supplementary information

Supplement A

Supplement B

Supplement C

## Data Availability

No new data were created or analyzed in this study. Data sharing is not applicable to this article.

## Glossary

**Active impacts**: ongoing injury to target resources. These can be sudden or unexpected, or slow to detect, but in any case, active impacts preclude pre-assessment or preemptive actions. Active impacts can have both immediate and delayed effects.
**Alert**: the act of becoming aware of the presence of an active impact or the expectation of an imminent impact. Some environmental disasters have alert systems built in (e.g., tsunamis and tornados) but most will rely on tracked information (e.g., weather forecasts) or observations from the field. The alert does not necessarily immediately follow immediate impacts as may be the case for a remote disaster that only becomes known after subsequent expansion or intensification of effects or after delayed observations of initial impacts.
**At-risk sites**: sites that are identified as being at elevated risk for incurring damage from an imminent impact.
**Decision Team (DT)**: the core administrative unit composed of key personnel from regulatory, management, and researchers who are responsible for making crucial decisions, setting goals, coordinating all activities and communications, and administering all aspects of an emergency response (including finances and project documentation).
**Delayed effects (subset of Active Impacts)**: effects of an active impact that follow the immediate effects after some time lapse. Two types of delayed effects include: 1) expansion or intensification of known impact effects (more of the same existing effects), and 2) secondary unanticipated effects (of an entirely different character from already observed effects).
**Extreme climatic events**: statistically rare climate-based events capable of inflicting dramatic ecological impacts (IPCC 2012; Zabin et al., 2022).
**Environmental disaster**: a catastrophic event that injures the natural environment. Here, the injuries are specific to a target resource and the disaster may be directly (oil spill) or indirectly (climate change) related to human action or independent of human action (events that are natural but devastating due to other human impacts such as habitat reduction).
**Field Team (FT)**: implementation personnel who are responsible for carrying out all field, lab, and husbandry responsibilities under the direction of the Decision Team. There is generally a lead team member who serves as a liaison to the Decision Team and who is responsible for FT oversight when implementing actions. The FT may consist of one team that works together throughout a response or may be composed of multiple teams charged with different responsibilities or with similar responsibilities at various locations.
**Grace period**: a period of time after the initial alert during which imminent impacts are expected. This period of time offers a respite during which pre-assessment and/or preemptive collections can be conducted if deemed appropriate and logistically possible.
**Immediate effects (subset of Active Impacts)**: the initial effects that happen during an active impact.
**Imminent impacts**: in some cases, conditions may exist that place a target resource at high risk for adverse future effects. In these cases, “imminent impacts” are anticipated and may merit and allow safe action in advance of expected changes, such as pre-assessments and/or preemptive collections.
**Impact**: direct or indirect effects inflicted on natural environments that can cause adverse consequences to the target resource. Direct effects described in CS1 and CS2 are burial of animals. Indirect effects include scouring and the removal of algae upon which black abalone feed.
**Impact Sites**: individual areas that are negatively affected by the environmental disaster, either as part of the initial footprint, or as part of an expansion of affected areas before the end of the environmental disaster. The impact site(s) may be the same as the impact zone (if the entire at-risk area is impacted) or there may be a mosaic of impacted and unimpacted sites nested within the larger, all-encompassing impact zone. Example: in a patchy fire, each burned patch is an impact site and the outermost perimeter that encompasses all patches is the impact zone.
**Impact Zone**: the impact zone encompasses the most inclusive extent of the environmental disaster, including unaffected areas between impact sites. If there is only one impact site, an impact zone and impact site are the same. However, an impact zone may consist of multiple, disjunct impact sites, separated by unaffected sites. Over time, some unaffected areas may be lost as adjacent impact sites expand and coalesce into a larger, contiguous impact site. Other unaffected sites may persist as such through the end of the environmental disaster. Example: in a patchy fire network, a perimeter drawn to encompass all patches of the fires outlines the impact zone while each individual fire patch is an impact site.
**No action**: one option in response to an active impact. Here, the DT opts to do nothing (other than possible remote monitoring) either because safety is at an unacceptable level, other logistics preclude action, or because a cost/benefit analysis suggests that no or delayed action may improve the overall outcome. No action also may be the preferred option in advance of delayed effects.
**Pre-assessment**: this action is possible only in the case of imminent events. A pre-assessment is carried out in an area where impacts are expected to occur in the future, allowing scientists to gather data about the habitat, population, and conditions present prior to an impact from a disaster. This information can influence decisions about conducting preemptive collections and may be used after any future impacts to inform injury assessment.
**Preemptive action/collection**: those that are initiated in an area not yet physically affected by a disaster but at high risk for imminent or delayed effects. These actions/collections are done in anticipation of event-related impacts and can occur either prior to any impacts at all (imminent impacts) or in areas adjacent to an impact site that are expected to be affected by expanding impacts beyond the initial impact footprint. Preemptive collections may accompany pre-assessment (imminent impacts) or reconnaissance (active impacts). However, in especially urgent situations, preemptive collections may be conducted without any ancillary data collection to expedite and maximize the extent of collections.
**Reactive action/collection**: those that are initiated in areas where the environment has already been affected. The locations and timing of reactive actions/collections may expand as impacts expand beyond the initial footprint of the impact site. Reactive collections may be done in tandem with reconnaissance if time and other logistics allow. However, in especially urgent situations, reactive collections may be conducted without reconnaissance to expedite and maximize the extent of collections.
**Reconnaissance**: allows scientists to gather data about the habitat, population, and conditions that exist after an impact has occurred (active impact). Data can be collected in the impacted site(s) to document effects to the target resource and in the adjacent areas to assess the risk of expanding impacts that follow. The data gathered during reconnaissance can be used to guide DT decision-making.
**Responsible party**: an entity responsible for the costs associated with an environmental disaster. Responsible parties in the U.S. after oil spills are the identified owner or operator of the facility from which oil originates. Most environmental disasters will lack a responsible party to fund the response and require rapid funding to proceed.
**Return**: releasing collected individuals to the same location from which it was collected. This may be possible in environmental disasters with rapidly remediated effects.
**Target resource**: the organisms, communities, habitats, ecosystems, or ecosystem functions of concern to natural resource managers.
**Translocate**: releasing collected individuals to a new location than that from which it was collected. This may be necessary after disasters that impart long-lasting or dramatic changes that are not quickly remediated or when there is a continued risk for expanding or intensifying effects.
**Unconsolidated material**: in this paper, unconsolidated material includes the mud, sand, soil, rock (from gravel to bus-sized boulders), vegetation (alive or dead, burned or unburned), man-made materials, water, and anything else that is carried downslope during a landslide or debris flow (mudslide). Once deposited, unconsolidated material forms a cement-like deposit that can take months to years to loosen and move.
