# Supplementary material for: Decision-making framework for response and management of environmental disasters (FRaMED)
Source: Environ Manage. 2026 Jun 19;76(7):219. doi: 10.1007/s00267-026-02502-4 (PMC13282301; doi:10.1007/s00267-026-02502-4)
Supplement: Supplementary file 2 — Supplement B [file 267_2026_2502_MOESM2_ESM.docx]

**Manuscript Information:**

- **Title:** Decision-making Framework for Response and Management of Environmental Disasters (FRaMED)
- **Journal:** Environmental Management
- **Authors:** Bragg, Wendy K.*, Bell, C.A., and Lonhart, S.I.
- ***Corresponding author information:** wbragg@ucsc.edu; University of California Santa Cruz

**Field Reconnaissance Assessment Form**

Black Abalone Emergency Response (non-oil spill emergencies)

**Instructions:** This form is to be used during a reconnaissance trip to record information pertinent to the subsequent debriefing.

***Notification must be sent to [regulatory agency] prior to operation in case rescue is initiated***

| **Incident Name:** |  |
| --- | --- |
| **Date/s:** |  |
| **Team Members Full Names:** |  |

| **Photos taken?**  Y* / N | **Drone flights?**  Y* / N |
| --- | --- |

*attach as many Photo Log and Drone Flight datasheets as needed

**Time on site:** __________ to __________  **Low Tide:** __________(ft) at __________(hr)

**Weather and Sea Conditions** (affecting quality of sampling) (use codes listed below)

**Swell/Surge:** 0 / L / M / H **Wind:** 0 / L/ M / H **Rain:** Y* / N **Recent Rain:** Y* / N

*detail below in Notes on physical conditions

**Notes on Physical Conditions:** ____________________________________________________________________________

____________________________________________________________________________

____________________________________________________________________________

**Safety Reminders:**

- **Safety of personnel is always the top priority.**
- **All Field Team Members (not just the Field Lead) are charged with assessing site safety and should immediately share any concerns with Field Lead and/or personally discontinue operations if safety becomes a concern.**
- **Safety assessments should be done immediately upon arrival (prior to coastal access) and frequently while at the site to ensure the safety of all personnel.**
- **Be aware of the potential for changing safety conditions with expanding/intensifying/changing site conditions.**
- **When in doubt, discontinue operations and retreat to a safe area.**

**LOGISTICS**

| **Site Access** Information  [i.e. private land access permissions, gates, gate codes, any issues accessing site (e.g., interactions with public, land managers, site conditions), etc.] |
| --- |
|  |
| **Planning** Information  [i.e. GPS locations, driving directions, parking, hiking pathways, ropes needed, etc., required tide heights for access, point of contact (POC) information, preferred method of contact, POC expectations for access, POC restrictions on access] |
|  |
| **Safety** Information  [i.e. Is the site safe? If impacts expand/intensify/change will the safety conditions change? Is additional safety gear/planning needed?] |
|  |

**ABALONE POPULATION ASSESSMENT**

| **Density** of abalone at the site  If AHA style surveys are conducted, list the surveys completed (i.e. # segments, extent, any deviations/additions to the standard protocol such as sediment collections)  **Reminder to record removability “Y/N” by individual when conducting AHA** |
| --- |
| Example: “Team #1 completed 5@10m AHA segments AHA upcoast, team #2 completed 5@10m AHA segments downcoast…” |
| **Observed impacts to abalone**  Record the **Extent** of the impacts (estimate of the proportion of population impacted) and the **Intensity** of the impacts (mild, moderate, severe, in relation to existing population) |
|  |

**HABITAT ASSESSMENT**

| **Composition/Nature** of the impacts  [i.e. composition of materials, sources, sediment, debris, burned debris, etc.] |
| --- |
|  |
| **Intensity** of the impacts  [i.e. mild, moderate, complete inundation. Detail variations across the site] |
|  |
| **Extent** of the impacts  [length of coastline or area impacted, or reference drone mapping if completed. If applicable, observe adjacent areas such as burn scars] |
|  |

**PREDICTIONS**

| Expectations for ameliorating or intensifying conditions and the predicted extent of coastline (m or km) to be at future risk |
| --- |
|  |
| How many abalone, or what percentage of the abalone population, is predicted to be impacted? |
|  |
| What extent of habitat may be impacted if the situation expands, continues, or intensifies? Consider the impact to different habitat quality types (good, moderate, poor)  Consider adjacent areas such as burn scars and proximity to freshwater outflows |
|  |
| **Removability** of at risk abalone- what proportion/#s of abalone can be rescued. Split into discrete groups if predictions can be made for multiple stages of rescue. This information may also be gleaned from AHA surveys if conducted. |
|  |
| **Methods/Tools** needed for rescue- what methods should be used and what tools may be needed to conduct rescue? |
|  |
