# Supplementary material for: Decision-making framework for response and management of environmental disasters (FRaMED)
Source: Environ Manage. 2026 Jun 19;76(7):219. doi: 10.1007/s00267-026-02502-4 (PMC13282301; doi:10.1007/s00267-026-02502-4)
Supplement: Supplementary file 3 — Supplement C [file 267_2026_2502_MOESM3_ESM.docx]

**Supplementary C: Overarching Considerations**

Five key principles that underlie every aspect of disaster response planning and operations but are especially and critically important during the Implementation Phase include:

- Safety
- Communication
- Cost/benefit analysis
- Asset awareness
- Flexibility

*Safety* is the responsibility of all personnel and should remain central at all times: from preparation to implementation. Organizationally, it is likely that FT personnel will be drawn from multiple institutions with varying degrees of oversight, limitations, and risk management. The DT must be cognizant of this variability and ensure that all members are covered, as appropriate to the locale, by their institution. Important considerations may include liability, medical, and safety regulations. Once a response is underway, safety becomes even more important. Safety assessments must occur early and often and will inform potential actions, particularly if conditions worsen. All team members (DT and/or FT) should be empowered to call attention to unsafe conditions and to halt an operation if needed.

*Communication* plans should be clearly outlined by the DT to address internal (between team members, whether FT-to-FT, FT-to-DT, or DT-to-FT) and external (with the public or media) communication. An internal communication plan should clearly address expectations for how and when the FT(s) should contact the DT for regularly scheduled check-ins. The DT should also develop external communication guidelines for when, how, what, and by whom information will be released to the public. External communication guidance is especially important if there is any sensitive information that needs to be protected.

*Cost/benefit analyses* will be at the center of each decision-making step. Both financial/logistics and ecological/environmental costs must be balanced against the benefits of action. Moreover, environmental and ecological constraints must be considered. For instance, varying degrees of injury and mortality will likely accompany collection of individuals; these “certain” losses should be factored into decision-making considerations. Ultimately, ecologically-informed decisions rest on finding a balance among certain costs (injury, mortality inherent in collections), the risks and potential costs associated with leaving the resource *in situ*, and the certain and/or uncertain benefits of collections.

*Asset awareness* includes identifying and leveraging operation-specific assets to maximize advantages and limit disadvantages. It is unlikely that all assets will be known in advance of an alert, so team members should be encouraged to search for assets in unusual places and to communicate identified assets to the DT. Examples include unique talents of team members, community support, and developing site conditions. For instance, if working in advance of an approaching fire, forecasted cool, calm weather could offer a window of time to access at-risk sites preemptively.

Finally, *flexibility* is essential to an adaptive and effective operation. FTs may need to diverge from initial plans of action, which may in turn necessitate a discussion with the DT. The DT may opt to grant experienced FTs a degree of latitude to adjust planned actions *in* *situ* (for instance, deciding during reconnaissance that reactive collections should be initiated immediately rather than waiting to brief the DT of observations and then returning later to do possible collections) while reserving more oversight for less-experienced FTs (for instance, requiring a check-in call before shifting actions). The decisions by the DT and actions of the FT reflect an ongoing, dynamic, and potentially iterative process placing safety first, and relying on effective communication and personnel flexibility to achieve an efficient and effective outcome. See Supplementary (A) Decision Guidance for more thorough consideration of topics to guide goal-setting and communication.
